# Effect of manual and digital contact tracing on COVID-19 outbreaks: a study on empirical contact data

**DOI:** 10.1098/rsif.2020.1000

**Published:** 2021-05-05

**Authors:** A. Barrat, C. Cattuto, M. Kivelä, S. Lehmann, J. Saramäki

**Affiliations:** ^1^ Aix Marseille Univ., CNRS, CPT, Turing Center for Living Systems, Université de Toulon, Marseille, France; ^2^ Tokyo Tech World Research Hub Initiative (WRHI), Tokyo Institute of Technology, Tokyo, Japan; ^3^ Computer Science Department, University of Turin, Turin, Italy; ^4^ ISI Foundation, Turin, Italy; ^5^ Department of Computer Science, Aalto University, Aalto, Finland; ^6^ Technical University of Denmark, Copenhagen, Denmark

**Keywords:** COVID-19, contact tracing, temporal contact networks

## Abstract

Non-pharmaceutical interventions are crucial to mitigate the COVID-19 pandemic and contain re-emergence phenomena. Targeted measures such as case isolation and contact tracing can alleviate the societal cost of lock-downs by containing the spread where and when it occurs. To assess the relative and combined impact of manual contact tracing (MCT) and digital (app-based) contact tracing, we feed a compartmental model for COVID-19 with high-resolution datasets describing contacts between individuals in several contexts. We show that the benefit (epidemic size reduction) is generically linear in the fraction of contacts recalled during MCT and quadratic in the app adoption, with no threshold effect. The cost (number of quarantines) versus benefit curve has a characteristic parabolic shape, independent of the type of tracing, with a potentially high benefit and low cost if app adoption and MCT efficiency are high enough. Benefits are higher and the cost lower if the epidemic reproductive number is lower, showing the importance of combining tracing with additional mitigation measures. The observed phenomenology is qualitatively robust across datasets and parameters. We moreover obtain analytically similar results on simplified models.

## 1. Introduction

The coronavirus disease (COVID-19) epidemic was declared a pandemic by the World Health Organization on 11 March 2020. As of mid-March 2021, COVID-19 has reached at least 120 million confirmed cases worldwide and caused at least 2.6 million deaths [1].

In the first half of 2020, faced with an exponentially growing number of cases and given the absence of effective pharmaceutical treatment and lack of vaccine, governments have first had to rely on broad, nationwide measures to reduce the mobility and number of contacts between individuals, starting with school closures and eventually lockdowns of whole countries [2–5]. These non-pharmaceutical interventions (NPIs) have succeeded in limiting contagion [6] and they have been gradually lifted in many cases after the first wave. However, the building of population immunity has been slow [7], and new epidemic waves have occurred. Overall, the situation appears under control in only a few countries, and many are experiencing successive ‘waves’ and re-emerging outbreaks, with a corresponding heavy burden on the healthcare systems and renewed regional or nationwide lockdowns. Exit strategies after the end of a lockdown are thus clearly needed to avoid new resurgences until vaccination has reached sufficient fractions of the population.

General measures to reduce transmission risk have remained in place or have been lifted and then re-imposed. These include enhanced hand hygiene, mandatory mask wearing in a number of contexts like public transportation, shops or even whole cities and limits to the size of social gatherings. These measures are beneficial and very useful instruments and have in part mitigated the successive waves of the COVID-19 pandemic [8]. They are also much easier to enforce than regional or country-wide lockdowns and they have smaller economic costs. However, these measures have proven to be insufficient on their own. They are also still indiscriminate, as they are imposed on infected and non-infected individuals equally. Targeted measures, on the other hand, focus on stopping the spread where and when it occurs. The most common targeted intervention consists in the isolation of infectious individuals, which prevents the infected individual from further spreading the disease. This can be fully effective only if all infectious individuals are identified *before* they infect others (e.g. through symptoms or testing). However, in the case of COVID-19 a large fraction of transmissions occur before symptom onset [9–13]. Furthermore, many infected people develop only mild symptoms or no symptoms and are therefore typically not tested, despite being able to transmit the disease [12,14]. A natural way to cast a wider net around an identified case is the so-called *contact tracing* process, which endeavours to identify people who have been in long enough contact with an infectious individual [15,16]. The rationale is that those individuals, even in the absence of symptoms, might be infectious as well and that quarantining them and monitoring their health can both protect them and block further transmission.

Contact tracing is traditionally carried out via interviews of identified cases, followed by phone calls to the identified contacts to warn them and to ask them to go into quarantine [15,16]. Such ‘manual’ contact tracing (MCT) is labour intensive; it can be slow, and it critically relies on the ability of individuals to remember and identify their contacts. While this is easy in some contexts, such as households, our actual ability to recall and identify close-range proximity contacts is known to be limited. Retrospective surveys have found that brief contacts have a lower probability of being recalled and that contact durations are overestimated [17]. Moreover, in important contexts such as public transportation, shops or lifts (elevators), we often find ourselves in the proximity of unknown persons. In such cases, digital proxies for close-range proximity are currently viewed as a complementary and scalable approach, known as *digital contact tracing* (DCT), that could overcome the above limitations [18–20].

The broad adoption of smartphones has provided an opportunity to develop apps that use short-range device-to-device communication between phones to sense the proximity of their owners. The exchange of low-power Bluetooth packets between smartphones can indeed be used to detect proximity relations between persons whose phones are running the app: whenever a person who has installed a contact tracing app is diagnosed as infectious, a warning can be sent to all app users that the person has been in close proximity with during the previous few days [18,21,22]. Note that the proximity relations detected by Bluetooth are approximate and partially depend on the environment and detection thresholds that need to be set after calibration [21,23]. The wide dissemination of Bluetooth, however, currently makes it the best available proxy for physical proximity in DCT, and different technical architectures for DCT have been proposed, including so-called ‘decentralized’ solutions [21] that by design minimize the amount of information that is collected and shared. Exposed individuals warned via DCT are asked to contact the responsible health authorities and to quarantine themselves. Importantly, contact tracing apps do not rely on individuals recalling or naming their contacts nor do they require knowledge of their identity. However, the efficiency of contact tracing apps is obviously limited by their numbers of users, since both the infectious person and their contacts need to have installed the app, and by the compliance of alerted users. Some studies [22,24] have proposed to further extend the reach of DCT by using information on individuals situated two hops away from a confirmed case, along the digitally sensed proximity network (so-called ‘recursive’ contact tracing). This might however have privacy implications, and it is important to note that the success of app-based contact tracing depends on a complex interplay of proximity tracing technology, citizens’ trust and adoption [25], effectiveness of the exposure notification strategy and good integration with traditional contact tracing and with other public health capabilities and processes.

Several theoretical studies have investigated the potential effectiveness of proximity tracing apps in the fight against COVID-19 [15,16,18–20,22,26–30]. The studies that have most influenced the public discussion around contact tracing apps are based on a simplified, macro-level mathematical model that assumes homogeneous mixing of the population [18,31]. However, real social and contact networks are highly non-homogeneous and exhibit many non-trivial structural and temporal features, such as heterogeneous distributions of contact durations [17,32,33], sets of ties with correlated activities [34] and intermittent communities and dynamical social structures on multiple time scales [35,36]. Heterogeneities in contact structure and durations are known to be highly relevant for epidemic spreading processes, as well as group structures [37,38]. In particular, they have been shown to influence the estimation of the epidemic size for simplified models of infectious disease dynamics [39,40]. However, it is at this stage unclear whether the estimation of an intervention’s efficiency is impacted by these complex structural and temporal properties of real contact patterns: in particular, the dependence of an intervention’s effect on its parameters (here, for instance, the efficiency and speed of contact tracing or the adoption rate of the digital tracing app) might *a priori* differ in simple contact models and in real contact networks. Therefore, to go beyond homogeneous mixing results, some studies have considered artificial static network structures to study at a theoretical level the effect of heterogeneous structures [24], and other approaches have used large-scale agent-based models [27,30] or GPS data [20] to recreate synthetic daily aggregated networks of contacts and to evaluate the effectiveness of app-based contact tracing in more realistic settings.

Using high-quality empirical data on close-range proximity between individuals is thus an additional crucial step to deepen our theoretical understanding of contact tracing, understand the phenomenology of contact tracing efficiency in real-world settings and thus improve the grounding of the discussion on the relative and combined efficiency of MCT and DCT. State-of-the-art datasets of this kind were collected and made public over the years by several research efforts, in particular by two independent collaborations [33,41] who have used proximity sensors and smartphones in specific communities (such as among university students) and in highly relevant social contexts such as workplaces, schools and hospitals. These empirical data are temporally resolved and exhibit the complex structural and temporal complex features mentioned above [32,33,35,36,42]. They represent thus an important benchmark for realistic simulations of epidemic processes and interventions. We note here that these datasets were collected in non-pandemic situations. No such data are currently available to investigate how contact patterns have changed owing to restrictions and individual risk perception [43], and this has thus typically been modelled by effectively reduced transmission rates [4,29,30]. We consider here post-lockdown scenarios in which contact patterns and, behaviours could return to (almost) normal, and for completeness, we will also consider both reduced effective transmission rates as well as other ways to take such potential changes into account, such as random reduction of contacts or suppression of fleeting contacts. We also note that these data have been recently used [29] to derive parameters for the approach described in [18], and to determine—within that approach—which app-based contact tracing policies might be most effective.

Here, we use such empirical high-resolution contact network data to inform a compartmental model for COVID-19 [4] and to simulate directly the targeted measures described above in realistic, albeit circumscribed, contexts. In particular, we study the combination of app-based contact tracing and MCT, taking into account specific limitations of both approaches. We quantify the impact of interventions by measuring the reduction of the final size of the simulated epidemic. Moreover, we study the number of quarantines and the fraction of quarantined individuals who were not infected. We find that the mere isolation of symptomatic cases is not sufficient to substantially reduce the epidemic size, while interventions guided by contact tracing can have a strong impact. In particular, MCT, even when imperfect (i.e. limited recall of past contacts, delays in alerting contacts), yields a potentially strong reduction in epidemic size that grows linearly with the fraction of contacts correctly recalled. The effect of DCT, in turn, grows only quadratically with the fraction of app adopters, as expected from the constraint that both the case and the contact need to be running the app for a contact to be detected by their phones. Therefore, if DCT is used in isolation, a large fraction of adopters is required to obtain a large impact, although no threshold effects are observed: no specific adoption threshold exists that would lead the app to have no impact at a low level of adoption but suddenly become efficient if a certain level of adoption is reached. On the contrary, any degree of adoption yields a positive contribution. While the specific values of the epidemic size reduction due to contact tracing efforts depend on the parameters and contexts, the overall functional shapes are similar in all cases. In fact, we recover the overall qualitative behaviour of the epidemic size reduction in a simplified analytical model of propagation, showing the generality of this phenomenology.

We also observe that MCT and app-based contact tracing lead to similar numbers of quarantined individuals (which can be interpreted as the cost of the intervention) and fractions of non-infected quarantined individuals, for a given relative reduction in epidemic size (benefit of the intervention). In addition, even if the specific values obtained depend on parameters and contexts, this cost–benefit curve has a robust parabolic shape and exhibits a maximum: if contact tracing is effective enough, it might contain an outbreak early on, leading to few quarantine events, i.e. high benefit with low cost. We also provide analytical arguments to understand theoretically this robust parabolic shape.

Finally, we show that, for fixed app adoption and MCT parameters, recursive contact tracing further reduces epidemic size, at the cost however of a higher fraction of non-infected individuals among quarantined individuals. In fact, even at given benefit (epidemic size reduction), the cost in terms of number of quarantines and non-infected quarantined individuals is higher for recursive tracing than for standard tracing. While the data we use are limited to specific contexts, the robustness of the observed qualitative behaviour in various contexts and with respect to parameter changes, together with the fact that we can understand them using analytical approaches, hints at the generality of our results. We conclude that DCT and MCT complement each other, and their combination can help reach an optimal regime of high efficiency where the spread is suppressed at low cost in terms of quarantines. Importantly, DCT compensates for the inherent limitations in recall and scalability of MCT, but improvements in MCT efficiency can yield particularly strong effects and should thus be prioritized. Moreover, app adoption should clearly be as large as possible, but there is no specific level of adoption that must be achieved, as any improvement in adoption has a positive impact.

## 2. Results

**Figure 1.**
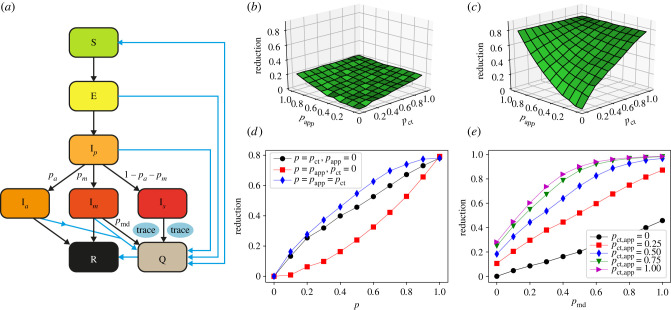
Model and impact of contact tracing on the epidemic size. (*a*) Schematic illustration of the investigated epidemic model. (*b*) Relative reduction in the average epidemic size as a function of MCT probability *p*_ct_ and app adoption rate *p*_app_, with respect to the situation of only case isolation, with *p*_md_ = 0.5, for OD, with *θ*_ct_ = *θ*_app_ = 15 min. Here $\tau_{\mathrm{ct}}=2\;\mathrm{days}$, *τ*_dct_ = 0. (*c*) The same for SD (threshold *θ*_ct_ = *θ*_app_ = 15 min). (*d*) Same as (*c*), for three slices: along the *p*_ct_ axis with *p*_app_ = 0, along the *p*_app_ axis with *p*_ct_ = 0 and along the diagonal *p*_ct_ = *p*_app_. (*e*) Relative reduction as a function of the testing and diagnosis rate of mildly symptomatic *p*_md_, for the SD with various values of *p*_ct_ = *p*_app_.

We model an outbreak of COVID-19 by means of a compartmental model [4] in which individuals can be in a series of discrete states describing the unfolding of the disease (see figure 1*a* and Methods for details and parameter values). Susceptible (healthy) individuals (S), upon contact with infectious ones, can contract the disease and first enter the exposed (non-infectious) state (E) and then a pre-symptomatic infectious state (I_*p*_). Pre-symptomatic individuals can remain asymptomatic during the whole infectious phase (I_*a*_), with probability *a*, or they can develop either mild or severe symptoms (I_*m*_ or I_*s*_), with respective probabilities *m* and *s* = 1 − *a* − *m*. The infectious state leads to recovery R (or death) after a typical time.

We simulate the model on empirical data describing time-resolved, close-range proximity interactions of individuals in two relevant settings: a community of students (SD, in the following) and an office (OD, in the following). The SD dataset describes the network of physical proximity among a population of more than 700 university students over a period of four weeks. The dataset was collected as part of the larger Copenhagen Networks Study [23,33], in which each participant was equipped with a smartphone and required to install data collection software. These devices were set to be permanently discoverable by Bluetooth and scanned for nearby Bluetooth devices at 5 min intervals (hence, the detected contacts have durations which are multiples of 5 min). Proximity was assessed by measuring the received signal strength (RSSI) of Bluetooth signals from nearby devices: a high RSSI means that the two devices are expected to be physically close, a low measure indicates that devices are further apart or that there are obstacles in between, although no precise relation between RSSI and distance can be assessed in general [44]. The OD dataset was collected in an office workplace staffed with about 200 people who agreed to wear proximity sensors for several days while in the workplace [41,42]; these sensors used low-power radio communication as a proxy for the close-range face-to-face proximity of individuals wearing the devices with a temporal resolution of 20 s [32]. Both the SD and OD datasets represent proximity interactions as a temporally ordered sequence of contact networks, where nodes are individuals and an edge between nodes indicates a proximity relation. The two datasets share both similarities and differences (see also Methods): owing to the face-to-face condition, the closer range and the smaller population size for the OD, the number of contacts registered per individual is much smaller; moreover, the 5 min temporal resolution of the SD means that the shortest possible contact lasts 5 min (20 s for OD), so that the contact times are larger for the SD than for the OD data. Despite these differences, similar complex temporal and structural features are observed [35]. Moreover, the different definitions of contacts in OD and SD could correspond to simulating different spatial ranges of disease transmissions and at-risk contacts for droplet-transmitted pathogens such as severe acute respiratory syndrome coronavirus 2 (SARS-CoV-2).

We run simulations starting from one initially infectious individual chosen at random, letting the epidemic evolve until no infectious individuals are present in the population. We first calibrate the rate of disease transmission from an infectious person to a susceptible person (see Methods) for each dataset to obtain a reproduction number *R*_0_ ≈ 3 in the absence of any containment measures [4,45]. Note that, because of the difference in the definition of a contact and in the temporal resolution of the two datasets, the transmission rate cannot be considered as the same for both datasets, but we instead fix *R*_0_ as the parameter defining the effective epidemic spreading capacity. We also considered simulations with lower *R*_0_ values, corresponding, for example, to re-emerging outbreaks during which a number of public health measures such as enhanced hygiene and mask-wearing are enforced to limit the spread, thus yielding an effectively lower *R*_0_: such measures are simulated by changing the transmission rate. Moreover, we considered as a possible result of behavioural change the removal of a fraction (25%) of contacts at random. As a baseline intervention, used as a reference to assess the relative improvement of contact tracing, we then considered a simple strategy that does not rely on any contact information: isolation (Q) of all cases with severe symptoms as well as isolation of a fraction *p*_md_ of the mildly symptomatic cases, until they recover. For *p*_md_ = 50%, this intervention yields a relative decrease in the average epidemic size of, respectively, 14% for the OD data and 26% for the SD data (lowering the average final epidemic size from 73% to 63% of the population in the OD and from 55% to 41% in the SD).

We then simulate MCT for all detected infectious cases (i.e. all severe cases I_*s*_ and a fraction *p*_md_ of the mild cases I_*m*_): for each case, an interview is assumed to be performed to identify all individuals who have been in contact with the case for an interval longer than *θ*_ct_ over the 48 h before detection. To take into account the limitations of MCT, we assume that only a fraction *p*_ct_ of the contacts are identified, and that a delay of $\tau_{\mathrm{ct}}=2\;\mathrm{days}$ takes place between the detection of a case and the quarantining of a recalled contact. In addition to MCT, we consider the possibility of DCT: if the identified case has a smartphone with a contact tracing app installed, a warning is sent to other app users who have been in proximity (as assessed by the contact data with the case for an interval longer than *θ*_app_ over the 48 h prior to detection). Since we assume that all such contacts are identified by the app, the key parameter for DCT is the fraction *p*_app_ of the population who has adopted the app. While we will mainly consider the value of *θ*_app_ set in the actual apps, we explore also larger values, which can be interpreted as what happens if a fraction of the actual contacts is not detected by the app, e.g. because of Bluetooth limitations. Moreover, we take into account potential delays due to possible app limitations (e.g. a limited number of exposure queries per day by the app) or to the time needed for an individual to self-isolate by introducing a delay *τ*_dct_ between warning and quarantine (*τ*_dct_ varying between 0 and 2 days).

The reduction in the average final epidemic size obtained by MCT and DCT, with respect to the situation of only case isolation, depends on the contact tracing parameters and efficiency. For simplicity we assume *θ*_ct_ = *θ*_app_ as these values are fixed by public health guidelines, and we vary the fraction *p*_ct_ of contacts identified by MCT and the fraction of app adopters *p*_app_ in the population. Note that, even if *p*_app_ in the general population is limited by the smartphone penetration, we consider here specific contexts where this penetration could be as high as 100% and we therefore explore the whole range of *p*_app_ values between 0 and 1.

Figure 1*b*–*d* shows how the reduction in epidemic size depends on these parameters. In particular, the effect is proportional to *p*_ct_ in the absence of DCT, while it grows quadratically with the fraction of app adopters in the absence of MCT. It is thus rather small at low app adoption and improving MCT leads to stronger effects. However, even in the hypothetical scenario of perfect MCT, DCT still improves the results owing to the shorter delay between detection and quarantine made possible by DCT, and the combination of DCT and MCT makes it possible to reach strong epidemic suppression effects even at intermediate parameter values.

A larger epidemic size reduction is observed for the SD case: this can be ascribed to the fact that the fraction of contacts lasting more than 15 min over 2 days (i.e. which can be traced by contact tracing) is much larger for SD (on average 59% of the individuals with whom a person has been in contact, corresponding to 96% of all the contact times) than for OD (on average 6% of the individuals with whom one has been in contact, corresponding to 45% of all the contact times). However, the qualitative behaviour of the epidemic size reduction is similar for both datasets, despite the differences in contexts and data collection methods. In the electronic supplementary material, we show that this behaviour is robust for other datasets and other parameter values (electronic supplementary material, figures S2–S8). To mimic a behavioural change, we consider in particular several reduced values of *R*_0_ obtained by a reduction in the transmission rate. Interestingly, we observe that the relative effect is larger for smaller values of *R*_0_ (electronic supplementary material, figure S5). Moreover, as the final epidemic size even without interventions is smaller for lower *R*_0_ (and the epidemic is also slower), the number of cases to which contact tracing needs to be applied is smaller, and thus the cost in terms of quarantines is also smaller. We also show in the electronic supplementary material the results obtained with datasets in which a fraction of contacts has been removed (electronic supplementary material, figure S6). We moreover investigate the sensitivity with respect to an increased value of the fraction of asymptomatics (electronic supplementary material, figure S7), and to reduced compliance of the users (electronic supplementary material, figures S2–S4).

Figure 1*e* illustrates the crucial role of detecting cases with mild symptoms. Even in the absence of tracing (*p*_app_ = *p*_ct_ = 0), increasing the probability of case detection increases linearly the reduction in the average epidemic size. This effect becomes even stronger when contact tracing is implemented and the combination of a large probability of detection and of contact tracing can make a strong suppression of the epidemic reachable. Finally, as shown in the electronic supplementary material, delays in the warning and quarantining of individuals using the app, as modelled by *τ*_dct_, slightly decreases the efficiency of the contact tracing (electronic supplementary material, figure S8), and different values of the thresholds *θ*_ct_ and *θ*_app_ lead to qualitatively robust results (electronic supplementary material, figures S2–S4).

**Figure 2.**
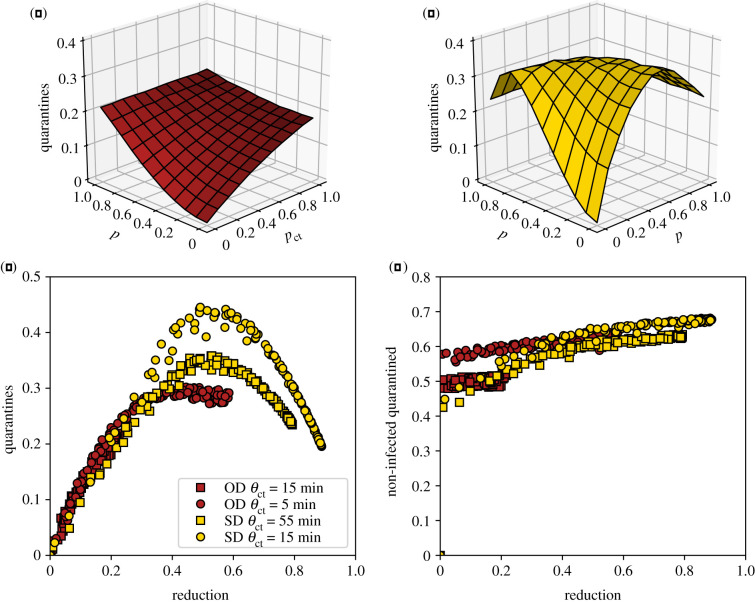
Number of quarantine events normalized by the population size. We show this number as a function of *p*_app_ and *p*_ct_, for (*a*) the OD data and (*b*) the SD data. (*c*) Number of quarantine events normalized by the population size as a function of epidemic size reduction, for the OD data (red squares; red circles for lower tracing threshold *θ*_app_ = *θ*_ct_ = 5 min), and the SD data (yellow circles; yellow squares for higher threshold *θ*_app_ = *θ*_ct_ = 55 min). (*d*) Ratio of non-infected quarantined individuals to quarantines, for the same datasets as in (*c*).

Contact tracing brings clear benefits as explored above, but also has a cost that can be quantified by the number of quarantine events in the population (note that a given individual might potentially be quarantined more than once). Moreover, a fraction of these quarantines are unnecessary, as some of the quarantined individuals are, in fact, healthy. Figure 2*a*–*c* explores this issue. The number of quarantine events, i.e. the cost of the intervention, initially grows with *p*_app_ and *p*_ct_, as expected. However, in some cases, this cost goes through a maximum and decreases for large enough DCT app adoption or MCT efficiency. This corresponds to the fact that a very efficient contact tracing procedure can actually stop an outbreak early on, so that the epidemic does not spread and few quarantines are necessary (this is indeed the case for large *p*_app_ and *p*_ct_ in the SD case, as seen in figure 1*c*). In fact, figure 2*c* shows cost–benefit curves of the contact tracing interventions, at fixed tracing parameters, i.e. the normalized number of quarantine events as a function of the reduction in average epidemic size. These curves all exhibit a typical parabolic shape, with a cost first increasing as a function of the benefit, reaching a maximum and then decreasing. Note that each curve puts together results obtained either by MCT, by DCT or by a combination of both: in other words, the cost depends on the benefit and not on the way (MCT or DCT) this epidemic size reduction was obtained. We also note on the other hand that the cost depends on the specific value of the threshold *θ*_ct_ = *θ*_app_ used to define at-risk contacts in the contact tracing procedure: lower thresholds make it possible to obtain higher benefit at large adoption or MCT efficiency (large *p*_ct_), but the maximal value of the cost, reached at intermediate adoption or MCT efficiency, is then larger. Finally, increased delays in contact tracing lead to slightly increased costs for a given epidemic size reduction (see electronic supplementary material, figure S9). This can be expected as delays mean that some of the individuals who have been in contact have time to become infectious and transmit the disease further, leading to a larger number of quarantines. On the other hand, very large values of case detection probability *p*_md_ yield both higher benefits (figure 1*e*) and lower costs (see electronic supplementary material, figure S10).

Figure 2*d* shows also the behaviour of the fraction of non-infected quarantined individuals. While this fraction depends only slightly on app adoption and MCT efficiency, it increases slightly overall as the epidemic reduction increases. This fraction also increases if the delay between case detection and contact quarantining increases, and decreases if the detection probability of mild cases *p*_md_ increases.

**Figure 3.**
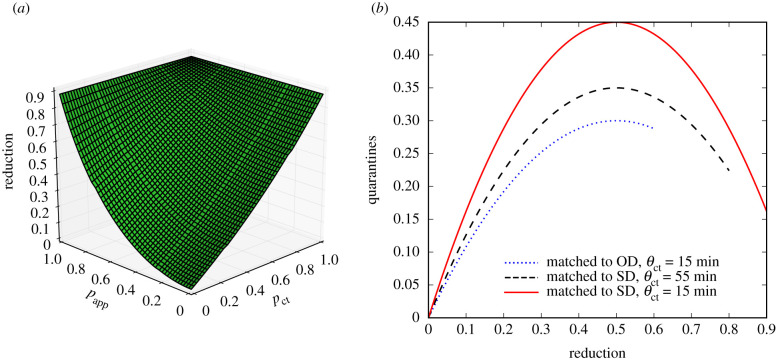
Results reproduced with simplified theoretical models. (*a*) Reduction in final epidemic sizes as a function of MCT probability *p*_ct_ and app adoption rate *p*_app_. For easier comparison with the simulation results, we have matched the parameters such that the epidemic sizes at *p*_app_ = *p*_ct_ = 0 and *p*_app_ = *p*_ct_ = 1 are the same as for the SD data shown in figure 1*c*. The overall shape of the surface is very similar, with MCT leading to an almost linear reduction while the app adoption leads to a convex shape. (*b*) Number of quarantine events normalized by the population size as a function of epidemic size reduction, obtained by the simple mathematical argument explained in the main text. The height of the curve depends on the parameters and the end of the curve is determined by the maximum reduction in epidemic size. For easier comparison with figure 2*c*, we matched the heights and the maximum reductions to those of the simulation results.

Our results are obtained with simulations of a compartmental model performed on state-of-the-art datasets describing human interactions, which include many complex features of such interactions, such as heterogeneities of contact durations, intermittent formation of groups, etc. Interestingly, we can actually obtain a very similar phenomenology using analytical arguments on simplified models. To this end, we consider a Susceptible–Infectious–Recovered (SIR) model on a static random network. Using a well-known mapping of SIR models to percolation problems [46], it is indeed possible to obtain an equation for the final epidemic size of such a process. In the electronic supplementary material, we show that it is possible to extend the percolation arguments to introduce both DCT and MCT, and we show in figure 3*a* that the resulting shape of the epidemic size reduction as a function of the tracing parameters *p*_ct_ and *p*_app_ closely reflects the phenomenology observed in our numerical simulations.

Moreover, the parabolic shape of the cost–benefit curves shown in figure 2*c* can also be explained by a simple argument involving two competing effects. (i) The more we want to reduce the epidemic size, the more we need to quarantine, and (ii) the more the epidemic size is reduced, the fewer quarantines are actually needed. In the absence of tracing, the epidemic size is not reduced but also no quarantines are being implemented (zero benefit and zero cost); at the other extreme the quarantines are done so aggressively that the disease can only infect a very small fraction of the population and the total number of quarantines (contacts of infected individuals) remains low as well. At the peak the quarantine rate is still low so that the disease spreads to a significant part of the population, but it is large enough so that many of the connections of the infected individuals are placed under quarantine.

The above argument can be formalized as follows. We denote the epidemic size reduction owing to contact tracing as *r* = 1 − *I*_*r*_/*I*_0_, where *I*_0_ is the epidemic size without quarantine measures and *I*_*r*_ is the epidemic size with the measures in place. First, as a mean-field assumption, we express the number of quarantines *q* as simply the product of the average number of contacts of a person during the infectious period *k*, the total number of infected people I_*r*_ and the quarantine probability *q*_*r*_, i.e. the probability that any contact with an infectious person leads to a quarantine: *q* = *q*_*r*_ *k*I_*r*_. Quarantines, however, yield a decrease in the epidemic size so that the number of infectious people is itself a decreasing function of the quarantining probability: I_*r*_ = *f*(*q*_*r*_)I_0_, with *f* a decreasing function. In other terms *r* = 1 − I_*r*_/I_0_ is an increasing function of *q*_*r*_. Figure 1*b*,*c* shows that the reduction in the epidemic size, *r*, is roughly linear with the fraction of people asked to quarantine owing to MCT, *p*_ct_. As the probability of a contact being quarantined *q*_*r*_ increases as well linearly on average when we ask more people to quarantine, this linearity is carried over to yield *r* ∝ *q*_*r*_. Inserting this as well as I_*r*_ = (1 − *r*)I_0_ into the above expression for *q* leads to *q* ∝ *r* − *r*^2^, i.e. a downwards opening parabola very similar to figure 2*c*, as shown in figure 3*b*. More details and examples are provided in the electronic supplementary material.

**Figure 4.**
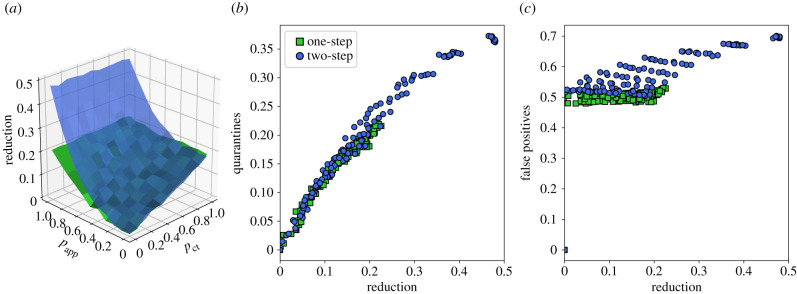
Recursive contact tracing versus single-step contact tracing. (*a*) Blue surface: epidemic size reduction as a function of MCT probability *p*_ct_ and app adoption rate *p*_app_ for recursive tracing (blue) and single-step tracing (green) for reference, for the OD dataset. *θ*_ct_ = *θ*_app_ = 15 min. (*b*) Normalized number of quarantine events versus epidemic size reduction for recursive (‘two-step’, blue) and single-step tracing (green). (*c*) Proportion of non-infected quarantined individuals for the same cases.

We finally show—in figure 4— the impact of a recursive DCT. At fixed *θ*_app_ and app adoption, the obtained reduction in the epidemic size is larger than with the normal app. The difference relative to the usual DCT is however quadratic in *p*_app_, meaning that this effect remains small unless app adoption is very high. Moreover, the resulting number of quarantine events is larger and depends more on the other parameters of the contact tracing. In fact, figure 4*b*,*c* shows that, at a given value of the epidemic size reduction, the number of quarantines is typically larger for the recursive DCT than for the usual DCT, and that the fraction of unnecessary quarantines becomes larger as well. In other words, the cost to obtain a given benefit is higher.

## 3. Discussion

In the fight against the COVID-19 pandemic, non-pharmaceutical interventions play a crucial role [19]. Moreover, in order to limit societal costs, targeted measures such as isolation of cases, contact tracing and quarantining of contacts are considered as essential in containing potential re-emergence of outbreaks [8,13,14,16]. As traditional MCT is labour intensive and limited by people’s ability to correctly remember contacts, app-based DCT is seen as a potentially useful complement and has been deployed in several countries [18,21,47]. Its actual efficiency, however, has been debated, in particular with respect to the level of adoption needed for it to make a difference [18,29,30,47,48].

In this study, we have provided new theoretical and qualitative understanding of the relative and combined efficiency of MCT and DCT by leveraging state-of-the-art datasets describing contacts between individuals in different settings, namely among office workers and among students of a university. Indeed, most previous works have considered either a homogeneous mixing hypothesis or model networks of interactions, while real contacts are known to display a wealth of structural and temporal heterogeneities having an impact on epidemic propagation. Even if the available contact network datasets correspond to limited contexts and populations, they do encompass all these heterogeneities. Their use in simulations is therefore a crucial step to consolidate our theoretical and practical understanding of the mechanisms of various interventions and, here in particular, to understand how their impact depends on the contact tracing parameters. Here, we have considered a compartmental model drawn from the recent literature on the propagation of the SARS-CoV-2 pathogen and simulated several mitigation measures, focusing on the reduction in the final average epidemic size in the population. The isolation of severe cases and of a fraction of mildly symptomatic cases, although beneficial, is not enough to contain the spread as pre-symptomatic individuals are known to be infectious, and as a fraction of infectious are moreover asymptomatic. We have then simulated contact tracing, both manual (MCT) and digital (DCT), and shown that both can yield a strong decrease in the final epidemic size. The relative impact of contact tracing is actually larger for lower reproduction numbers, and its cost lower: lower *R*_0_ means indeed a slower epidemic reaching fewer individuals even without interventions, so that contact tracing needs to be applied to fewer cases, and fewer people need to be quarantined to mitigate the epidemic (this is in agreement with the results of [30]). Contact tracing thus becomes more efficient in a situation where the epidemic is already partially mitigated by other measures that keep *R*_0_ as low as possible, showing the importance of combining contact tracing with other measures such as masks or hand hygiene, and that contact tracing might lose efficiency at too large *R*_0_. The epidemic size reduction moreover depends on the efficiency of the manual tracing, quantified by the fraction of contacts recorded for each detected infectious individual, and on the app adoption in the case of DCT. We find that this reduction grows linearly with the efficiency of MCT but only quadratically with the app adoption, as both case and contact need to have the app installed for a contact to be detected. We have shown that, at a qualitative level, this overall behaviour can be recovered analytically in a simplified epidemic model. We note that, as our study focuses on specific contexts (offices and university), the app adoption could potentially reach 100% in the corresponding populations, leading in that case to a very strong suppression of the epidemic. We have also shown that the cost of the intervention, as quantified by the number of quarantines, initially grows as the MCT efficiency and app adoption increase, but can become smaller if these parameters are high enough so that the epidemic is very efficiently contained. The cost–benefit curves show a typical parabola shape that can be understood using simple arguments: for low efficiencies of contact tracing, the cost increases with the reduction of epidemic size, but if the efficiency becomes large enough, the strong suppression of the outbreak leads to fewer cases, thus fewer contacts and fewer quarantines. We have directly shown how a simple analytical argument recovers this parabolic shape of the cost–benefit curves.

We also note that the DCT we have simulated does not imply knowledge of the contact network of infectious individuals, but simply that their contacts receive a warning and quarantine accordingly. This confirms that it is possible to develop strongly privacy-preserving protocols [21] that might reach large app adoption levels and thus yield a strong impact. Furthermore, we have shown that recursive DCT yields an increased impact, which, however, also grows only quadratically with app adoption. The added benefit thus remains small except at very high adoption, and comes at an increased cost in terms both of quarantine events and of non-infected quarantined individuals. Moreover, it is important to remark that recursing the contact tracing process over contacts of an index case entails building an explicit representation of the two-step networks of confirmed cases, exposing significantly more network information about those individuals than regular contact tracing. This additional network information dramatically increases privacy risks, as it can be more readily used to match the contact graph around a given user to social network data from other sources (e.g. online social networks, mobile call networks, organizational networks, etc.), increasing the probability of re-identification. The resulting privacy concerns might lead to lower app adoption in the public, and thus potentially to an actual loss of efficiency of the DCT efforts.

Our study has a number of limitations which are important to make explicit. In particular, the data we use correspond to limited social environments (a university campus and a workplace) and we do not provide an overall general study that includes all possible multiple and differentiated contexts and their mutual interplay. These data moreover do not include interactions between the individuals participating in those studies and the general population. Nor do they include information on the precise location of the contacts, for instance whether these contacts occurred indoors or outdoors, which is known to be important in the COVID-19 context. In addition, the data were collected in pre-pandemic situations, and awareness of the spread as well as imposed restrictions have changed the behaviour of individuals in ways difficult to apprehend. Nevertheless, these datasets correspond to the current state-of-the-art in terms of data describing human interactions, and we have also considered several ways of taking into account in an effective way behavioural changes, from reduced reproduction numbers to filtering of a fraction of contacts. Moreover, one could expect that post-lockdown scenarios would rapidly lead to a return to pre-pandemic behaviour in terms of contact patterns. Overall, the datasets, even with these limitations, include complex features of real contact patterns, and it was thus important to understand the behaviour of contact tracing interventions in simulations on such data, and not only on simplified models of interactions. The results show a robust qualitative behaviour for data obtained in different contexts with different data collection infrastructures (inducing different spatial and temporal resolutions), in terms of both the epidemic size reduction and the dependency of the cost of the intervention on the MCT efficiency and on the app adoption. Moreover, our analytical approach gives us a theoretical understanding of the mechanisms behind this behaviour and shows its generality. A detailed quantification and prediction of the size of the effects would require more detailed models of specific (possibly large-scale) populations taking into account age stratification and other socio-economic factors, but important insights have already been gained by our theoretical approach.

For instance, an important point to emphasize is that, although the effect of DCT is only quadratic in the app adoption, there is no threshold effect: any increase in adoption leads to an improvement in the epidemic spread mitigation. This is actually also true for MCT, showing that the quality of interviews and any improvement in the ability to correctly find contacts of infectious cases brings an important benefit. Overall, DCT, which is also able to trace contacts between individuals who do not know each other, yields an interesting complement to MCT, and the combination of MCT and DCT is able to suppress outbreaks at limited cost if the app adoption and MCT efficiency are sufficiently high.

As the app adoption in the general population is necessarily limited by smartphone penetration, it is finally interesting to emphasize that this limitation does not necessarily hold in a specific context such as a workplace or a university. In fact, one could expect assortativity or group effects to induce a very high adoption in such specific contexts or populations. This could make it possible to very rapidly suppress outbreaks in these specific populations, at minor costs in terms of quarantines. A full investigation of such assortativity effects is an interesting direction for future work, and could lead to policy indications on how to best focus testing and MCT resources towards populations where app adoption is expected to be lower.

## 4. Material and methods

### 4.1. Data

We use state-of-the-art publicly available datasets describing contacts between individuals in different settings, with high spatial and temporal resolution.

— The SD dataset [23,33] describes the interactions of 706 students, as registered by the exchange of Bluetooth signals between smartphones, for a period of one month. Each participant in the study was equipped with a Google Nexus 4 smartphone (used as the primary phone) and required to install study-designed data collection software. For Bluetooth measurement, all devices in the experiment were configured to be permanently discoverable, and to scan for nearby Bluetooth devices at 5 min intervals. The devices measured and recorded the RSSI: a high RSSI means that the two devices are physically close; a low measure indicates that devices are further apart or that there are obstacles in between [44].
— The OD dataset was collected by the SocioPatterns collaboration, using an infrastructure based on wearable sensors that exchange radio packets, detecting close proximity (less than or equal to 1.5 m) of individuals wearing the devices [32]. The temporal resolution of the data was 20 s. The data were collected among 232 individuals in offices during two weeks in 2015 [42]. If the epidemic simulated on the data lasts more than the dataset duration, we simply replicate it [49] by restarting from the beginning of the dataset.
— We show in addition in the electronic supplementary material results obtained using data collected among more than 300 students in a French High School during one week by the SocioPatterns collaboration, i.e. with the same infrastructure as the OD data [17].

In the SD case, the average number of distinct individuals contacted by a random individual during 1 day (average degree in the daily aggregated network) varies between 4 and 47, with strong variations between weekdays and weekends.

In the OD case, the average daily degree does not reach such large values, being between 8 and 10 depending on the day.

Moreover, the fraction of links with weight above 15 min over a period of 48 h is 59% for SD, corresponding to 96% of the total contact duration, while it is only 6%, corresponding to 45% of the total contact duration, in the OD case.

We refer to [33,42,50] for a detailed description of the datasets.

### 4.2. Epidemic model

We consider the compartmental epidemic model described in [4], which was designed to describe the various stages of the COVID-19 disease.

Susceptible individuals (S) can become infected upon contact with an infectious one, and then enter a latent phase (E) of duration *τ*_*e*_. They then become pre-symptomatic (I_*p*_) during *τ*_*p*_. Pre-symptomatic individuals can then become either asymptomatic (I_*a*_) or develop mild or severe symptoms, entering, respectively, the compartments I_*m*_ or I_*s*_. This happens with probabilities *a*, *m*, *s* = 1 − *a* − *m*. Recovery occurs with rate *μ*.

The probability per unit time of a susceptible becoming infectious upon contact with an infectious with severe symptoms is *β*. On contact with a pre-symptomatic who later on develops severe symptoms, the probability per unit time becomes *r*_*p*_*β*, and it is *r*_*β*_*β* upon contact with an asymptomatic or an infectious with mild symptoms, or with a pre-symptomatic who then develops mild or no symptoms.

The parameter values are given in table 1. The value of *β* is adjusted in order to fix the basic reproduction number to a desired value in the absence of interventions. More precisely, we define *R*_0_ as the average number of secondary infections from an initially infectious seed taken at random. We perform 4 × 10^3^ simulations for each value of *β*, adjusting it until the desired value of *R*_0_ is obtained. For *R*_0_ = 3, this yields *β* = 1.37 × 10^−3^ s^−1^ for OD and *β* = 2.1 × 10^−5^ s^−1^ for SD. We have also considered other values of *β* in order to obtain lower values of *R*_0_ corresponding to the effect of restrictions such as mask wearing or enhanced hand hygiene.

**Table 1.** Parameters of the compartmental model, taken from [4].

parameter	value
*τ*_*e*_	3.7 days
*τ*_*p*_	1.5 days
1/*μ*	2.3 days
*R*_0_	3
*a*	0.2
*m*	0.72
*r*_*β*_	0.5
*r*_*p*_	1

We have also considered the case *a* = 0.4, *m* = 0.52.

### 4.3. Interventions

We consider here a series of targeted interventions, i.e. that aim at preventing further transmissions from infected individuals.

#### 4.3.1. Isolation

The first intervention consists simply in isolating infectious individuals once they are identified. This happens with all infected developing severe symptoms, as they will reach out to health services. Moreover, it can also happen for a fraction of infected developing mild symptoms. The probability that an individual with mild symptoms is identified as infectious is *p*_md_. Moreover, we take into account that the reaction to symptoms is not instantaneous by introducing a delay *τ*_to isol_ between the appearance of symptoms and the isolation. Upon isolation, an infectious stops having contacts and becomes unable to transmit the disease.

Pre-symptomatics and asymptomatics cannot be identified and therefore are not isolated.

#### 4.3.2. Manual contact tracing

When an infected individual (a ‘case’) is identified, they are interviewed by healthcare workers and asked to remember their contacts of the last $n_{\mathrm{keep}}\;\mathrm{days}$. The persons who have been in contact with the case for a cumulative time longer than *θ*_ct_ during these days are then warned and asked to quarantine for *τ*_*q*_ = 14 days.

The imperfection of MCT is taken into account by the following parameters:

— only a fraction *p*_ct_ of the contacts longer than *θ*_ct_ are recalled;
— only a fraction *p*_*c*_ of the contacts agree to quarantine; the others do not act;
— there is a delay *τ*_ct_ between the detection of the case and the quarantine of their identified contacts.

The health of the quarantined individual is monitored. Therefore, if a quarantined person develops symptoms (even mild), they are interviewed to trace their recent contacts, with the same procedure as the initial case. The parameter values are given in table 2.

**Table 2.** Parameters of the interventions. We also explore *p*_*c*_ = 0.6 and the whole range 0 ≤ *p*_md_ ≤ 1.

parameter	value
*τ*_to isol_	0.5 day
*p*_md_	0.5
*τ*_*q*_	14 days
*n*_keep_	2 days
*p*_*c*_	1
*τ*_ct_	2 days
*τ*_dct_	from 0 to 2 days

#### 4.3.3. Digital contact tracing

We consider, either by itself or in addition to MCT, the possibility that individuals have installed a privacy-preserving proximity-tracing app. The app registers proximity events with other individuals equipped with the app.

Whenever an app-adopter is diagnosed as infected (the case), the anonymous random identifiers used by their app in the last $n_{\mathrm{keep}}\;\mathrm{days}$ are uploaded to the central server (see [21] for details).

All app-adopters regularly check the central server and their app compares the list of identifiers of infected individuals with the list of identifiers received in the previous days. If the app detects that the cumulated time in contact with (one or several) infected app-adopters in the last $n_{\mathrm{keep}}\;\mathrm{days}$ exceeds *θ*_app_, it triggers a warning to contact health authorities and go into quarantine.

We assume that the transmission of information is instantaneous, but a delay *τ*_dct_ can take place between the warning of an individual by the app and the start of their quarantine. The other parameters are the fraction of app-adopters in the population, *p*_app_, and the probability *p*_*c*_ that an individual receiving a warning goes into quarantine. Non-compliant individuals do not change their behaviour. If a quarantined app-adopter develops symptoms (even mild), the process is iterated, i.e. their identifiers are uploaded. The parameter values are given in table 2.

#### 4.3.4. Recursive contact tracing

We finally implement recursive (two-step) warnings for app-adopters: in this case, we assume that a protocol is in place so that app-adopters can compute the cumulative contact time during the previous *n*_keep_ both with app-adopting infected individuals and with app-adopting contacts of app-adopting infected individuals who have received a warning by the app. If this cumulative time exceeds *θ*_app_, it triggers a warning to contact health authorities and go into quarantine.

The parameters are the same as for the DCT: the fraction of app-adopters *p*_app_, and the probability *p*_*c*_ that an individual receiving a warning goes into quarantine. Non-compliant individuals do not change their behaviour.

### 4.4. Quantification of the impact of interventions

For each scenario, defined by a given set of interventions with a given set of parameter values, we perform 10 000 numerical simulations with a single, randomly chosen individual in the latent phase at the initial time. Simulations are run until no infected individuals are present in the population, i.e. all individuals are either susceptible or recovered.

In order to quantify the impact of the spread on the population, and the effect of the various interventions, we measure:

— the average final epidemic size, i.e. the fraction of individuals who are recovered at the end of the simulation;
— the fraction of population going through quarantine;
— the fraction of the quarantined individuals who were in fact susceptible.

In particular, we quantify the effect of interventions by the relative reduction in the epidemic size, defined as $\frac{I_{0}-I_{r}}{I_{0}}$, where I_0_ is the average final epidemic size without interventions and I_*r*_ is the average final size in the presence of interventions.

## Data accessibility

We use only publicly available data. In addition, we have made our code available at https://github.com/jsaramak/contact_tracing and https://github.com/barrat/digitalcontacttracing.
